# How the Opinion of Others Affects Our Valuation of Objects

**DOI:** 10.1016/j.cub.2010.04.055

**Published:** 2010-07-13

**Authors:** Daniel K. Campbell-Meiklejohn, Dominik R. Bach, Andreas Roepstorff, Raymond J. Dolan, Chris D. Frith

**Affiliations:** 1 Wellcome Trust Centre for Neuroimaging, 12 Queen Square, London WC1N 3BG, UK; 2 Niels Bohr Project “Interacting Minds,” Centre of Functionally Integrative Neuroscience, Århus University, 8000 Århus, Denmark

**Keywords:** SYSNEURO

## Abstract

The opinions of others can easily affect how much we value things. We investigated what happens in our brain when we agree with others about the value of an object and whether or not there is evidence, at the neural level, for social conformity through which we change object valuation. Using functional magnetic resonance imaging we independently modeled (1) learning reviewer opinions about a piece of music, (2) reward value while receiving a token for that music, and (3) their interaction in 28 healthy adults. We show that agreement with two “expert” reviewers on music choice produces activity in a region of ventral striatum that also responds when receiving a valued object. It is known that the magnitude of activity in the ventral striatum reflects the value of reward-predicting stimuli [1–8]. We show that social influence on the value of an object is associated with the magnitude of the ventral striatum response to receiving it. This finding provides clear evidence that social influence mediates very basic value signals in known reinforcement learning circuitry [9–12]. Influence at such a low level could contribute to rapid learning and the swift spread of values throughout a population.

## Highlights

► Agreement with reviewers activates the same neural circuitry as object rewards ► Basic signals of a reward are modulated by social influence on its value ► Strongly influenced individuals produce more neural responses to disagreement ► The anterior insula cortex responds to unanimous opinions of others

## Results and Discussion

Of the few studies of social influence in the human brain [13–16], none have unambiguously shown the level of the value system at which social influence affects the value of an object when the object is received. Agreement between one's own opinion and normative opinion modulates activity in the ventral striatum [14], analogous to the dopamine-mediated reward signal observed in this region during reinforcement learning [9–12]. It has been proposed that changes in ventral striatum activity also predict subsequent conformity, but it had previously not been possible to distinguish such activity from that associated with social conflict [14]. We independently manipulated object reward, opinions of others about its value (social agreement), and their interaction in the same task and subjects. We delineated basic neural signals that track changes of object value caused by the opinions of others.

### Subjects and Ratings

**Figure 1:**
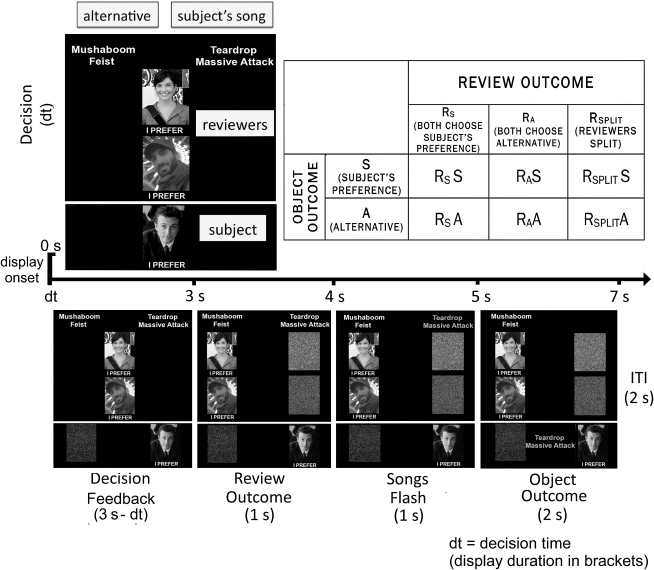
Task Displays, Timing, and Design Each trial began by the subject indicating his or her preference for either a song that the subject provided or an unrecognized alternative (by moving his or her picture beneath the preference). Songs choices (one on left, one on right) appeared above pictures of reviewers and the subject (aligned in the center) in white font. Pictures were black and white. Subjects pressed the left button to move their picture left or the right button to move it right. A scrambled picture of the subject was placed on the opposite side. Next, subjects learned the reviewer opinions. The picture of each reviewer was moved under his or her respective preference. A scrambled picture of each reviewer was placed on the opposite side. Finally, the songs flashed between white and green font and one song was chosen for the subject's token, which appeared at the bottom of the screen in green font. Review outcomes were independent of object outcomes. Subjects knew that the ten songs with the most tokens at the end of the task would be purchased for them. A 2 s intertrial display (not shown) was a fixation cross. In the 2 × 3 design (top right), the independent variables were *review outcome*: R_S_ (reviewers chose the subject's preferred song), R_A_ (reviewers chose the alternative), and R_SPLIT_ (split; one reviewer chose the subject's preferred song; the other chose the alternative); and *object outcome*: S (subject gained a token for his or her preferred song) and A (subject gained a token for the alternative song). These variables formed a 2 × 3 design matrix of six conditions: R_S_S, R_S_A, R_A_S, R_A_A, R_SPLIT_S, and R_SPLIT_A. The example shown corresponds to the R_A_S condition. See Supplemental Experimental Procedures for full task description.

One week prior to scanning, 28 healthy subjects (15 male, 13 female) submitted a list of 20 songs that could be purchased from an online music store and that they desired but did not yet own. On test day, each subject rated each song on a scale of 1 (“low”) to 10 (“high”) for desirability and read descriptions (and also viewed pictures) of two “expert” music reviewers before rating each reviewer from 1 (not at all) to 7 (very much) for how much they would trust that reviewer to pick a song that they (the subject) would like. Subjects then performed the task described in Figure 1 while being scanned with functional magnetic resonance imaging (fMRI). After scanning, subjects rated the songs and reviewers again. The mean song desirability rating was 7.4 ± 0.07 before the experiment and 7.61 ± 1.6 after the experiment. The mean reviewer rating was 4.43 ± 0.91 on a scale from 1 (“very unlikely”) to 7 (“very likely”) that the reviewer would choose a song that the subject would like). The mean male reviewer's rating was 4.61 ± 1.1, and the mean female reviewer's rating was 4.27 ± 1.1. Thus subjects perceived both reviewers as capable of choosing music that the subject would like.

To obtain a measure of influence for each subject, we carried out linear regressions to determine *B*_inf_: the number of standard deviations by which behavioral ratings of songs increased or decreased after the experiment with net reviewer opinion of that song (mean *B*_inf_ = 0.091, standard deviation [SD] = 0.17; see Figure S1 available online). Net reviewer opinion was the difference between the number of times that reviewers preferred the subject's song and the number of times that reviewers preferred the alternative.

### fMRI Results

Unless otherwise stated, all fMRI analysis was completed by using whole-brain cluster-corrected analysis with standard FMRIB software library (FSL) [17] default settings (cluster definition: Z > 2.3 and cluster probability threshold: p < 0.05). Peaks are specified as coordinates (x, y, z) in Montreal Neurological Institute (MNI) space (mm). A summary of all fMRI activations can be found in Table S1.

#### Object Reward

**Figure 2:**
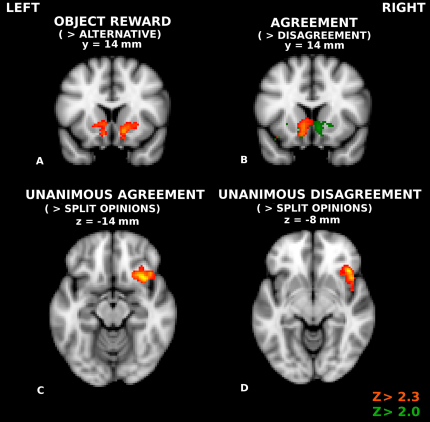
Main Effects (A) Object outcome [R_S_S + R_A_S] – [R_S_A + R_A_A]. Highlighted anatomy was more active when the participant received a token for his or her originally preferred song relative to receiving one for the alternative. (B) Review outcome [R_S_S + R_S_A] – [R_A_S + R_A_A]. Highlighted anatomy was more active when both reviewers agreed with the subject's preference compared to when they both preferred the alternative. Green maps show activation of the same contrast at a slightly reduced cluster definition threshold (Z > 2.0, p < 0.05). See also Figure S2 and Table S2. (C) Unanimous reviewer agreement [R_S_A + R_S_A] – [R_SPLIT_]. Highlighted anatomy is more active when both reviewers agree with the subject than when one chooses the subject's song and the other chooses the alternative. (D) Unanimous reviewer disagreement [R_A_A + R_A_S] – [R_SPLIT_]. Highlighted anatomy is more active when both reviewers disagree with the subject compared to when one chooses the subject's song and the other chooses the alternative. Unless otherwise specified, all activations are whole-brain cluster-corrected Z statistic maps (Z > 2.3, p < 0.05), which were overlaid onto the standard MNI brain. Coordinates of brain sections are indicated in MNI space (mm).

Subjects randomly received a token for one song on each trial (subject's preference or an alternative). Receiving a token for the preferred song compared to receiving one for the alternative elicited more blood oxygenation level-dependent (BOLD) activity in the ventral striatum (peak MNI coordinates [mm]: 14, 10, −8; −16 16, −2) (Figure 2A), left lateral prefrontal cortex (peak −46, 40, 4), and posterior cingulate cortex (peak −2, −36, 34).

#### Agreement with Expert Reviewers

Subjects also learned whether or not two reviewers shared their preference. Having the same preference as both reviewers elicited greater BOLD activity in left ventral striatum (peak −10, 8, −12) compared to when both reviewers preferred the alternative (Figure 2B). Slightly reducing the cluster definition threshold (Z > 2.0) revealed bilateral activation in the ventral striatum with a mirrored peak on the right (peak 8, 8, −12). This finding supports a role of the ventral striatum in processing of agreement with others [14] and extends this role to agreement with just two expert individuals. Analysis of the same contrast using a mask of object reward generates peak activation in the ventral striatum (peak −8, 10, −10), confirming that a rewarding object activated the same region as agreement with others.

What is reflected in a ventral striatum signal during agreement? In social contexts, learning to predict the mental states of others has been theorized to require activity in regions other than the ventral striatum (e.g., medial prefrontal cortex) whereas the ventral striatum tends to be more concerned with reinforcement (even if socially derived) [18–21]. When people share music taste, they allocate more rewards to each other, evaluate each other more positively, and are more likely to become friends [22–25]. Agreement with experts might also predict more rewarding choices in the future. Therefore, one possibility, given overlap with activation from object reward, is that subjects derived an associated reward from sharing preferences with reviewers.

Of note, seven participants reduced their subjective value of a song as the number of positive reviews of that song increased (negative *B*_inf_). Even these subjects produced a significant ventral striatum signal in a group analysis of agreement (right peak 6, 14, −6; left peak −6, 16, 2) (see Supplemental Experimental Procedures). This could mean that there was some low-level reward from sharing a preference with reviewers even if object values did not change to attain more of it. Alternatively, this could mean that ventral striatum activity reflected something other than reward such as changes in salience of songs and reviewers as a result of reviewers' preferences [26, 27].

Greater activity was also found along the ventral section of the parieto-occipital fissure and anterior calcarine sulcus (left peak −16, −56, 2; right peak 8, −62, 10). Activation extends into ventral posterior cingulate cortex (including retrosplenial cortex) and visual cortex. Additional activation was found in the occipital fusiform gyrus (V4) (peak −18, −86, −14). These activations are discussed in Figure S2 and Table S2.

**Figure 3:**
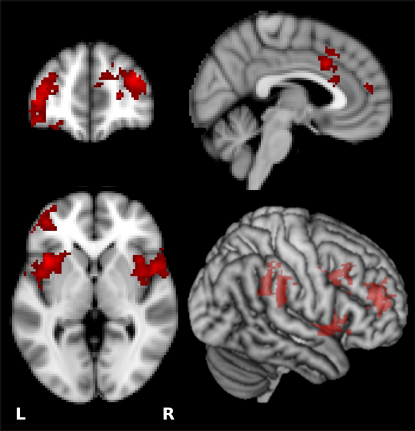
Disagreement with Others and Social Influence [[R_A_S + R_A_A] – [R_S_S + R_S_A]] × *B*_inf_. In the contrast of disagreement relative to agreement, the highlighted anatomy's activation varied between subjects with *B_inf._* The more an individual was influenced by reviewer opinions, the more insula cortex and/or central opercular cortex (right peak 52, 8, 2; left peak −38, 14, 0), dorsal anterior cingulate cortex (peak 4, 16, 34), and lateral prefrontal cortex (right peak 36, 48, 22; left peak −44, 48, 4) and right temporoparietal junction (TPJ) (66, −30, 36) activity was produced when he or she disagreed with the reviewer. Activations are whole-brain cluster-corrected Z statistic maps (Z > 2.3, p < 0.05), which were overlaid onto the standard MNI brain at coordinates (mm) 4, 48, 0. Search depth of overlay in 3D image is 8 mm from the surface.

Entering *B*_inf_ as a between-subject regressor in the above contrast showed that the more influenced a subject was by reviewer opinion, the more activation was found in the regions highlighted in Figure 3 during disagreement with reviewers. The right temporoparietal junction has been shown to monitor others' choices [18, 28] and, unsurprisingly, was more active in individuals that are more influenced by them. Lateral prefrontal cortex, also more active in this contrast, has been shown to be involved in reputation management [29] and social reasoning (see [30] for review). Findings that susceptibility to influence (*B*_inf_) correlated with dorsal anterior cingulate cortex and anterior insula cortex activity during disagreement replicate prior findings (see [13, 14] for discussion). These activations suggest that those who are influenced are more sensitive to conflict. However, they do not necessarily reflect social influence on object value. That effect is described next.

#### Social Influence on Value of Objects

**Figure 4:**
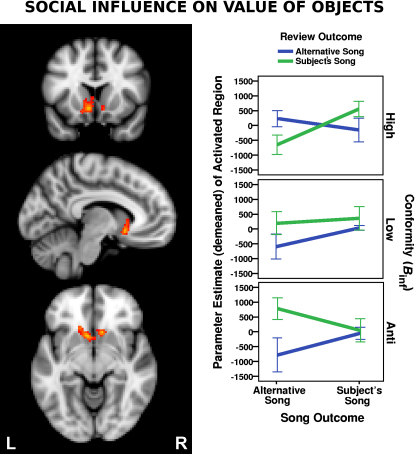
Social Influence on Value of Objects [[R_S_S – R_S_A] – [R_A_S – R_A_A]] × *B*_inf_. Subjects received their preferred song or the alternative after learning what reviewers preferred. The left panel shows the location of statistically significant reward activation due to social influence in the ventral striatum (400 voxels, Z_max_ = 3.44, right peak 10, 18, −8; left peak −6, 14, −8). The map results from the contrast of the interaction between review outcome and object outcome varying between subjects with *B*_inf_. Activations are whole-brain cluster-corrected Z statistic maps (Z > 2.3, p < 0.05), which were overlaid onto the standard MNI brain at coordinates (mm): −8, 14, −8. The right panels plot the mean parameter estimates (PEs) for five high-influence (most positive *B*_inf_), five low-influence (*B*_inf_ near 0), and five anti-influence subjects (most negative *B*_inf_) within the active cluster in the left panel (ventral striatum). The right panel is for illustration of the interaction only. This plot's standard error bars (±1) should be interpreted knowing that only five participants are indicated in each panel. Statistical inference should be made from the left panel and Table S1.

The magnitude of an outcome's value correlates with the magnitude of BOLD activity within ventral striatum [1–4]. These value signals can be subjective and alterable [5–8]. We therefore explored whether reviewer opinion of an object would influence value-dependent signals generated when receiving that object (i.e., whether reviewer opinion about a song token's value relative to its alternative modulates the value-related BOLD activity associated with receiving that token relative to receiving its alternative; [R_S_S – R_S_A] – [R_A_S – R_A_A]). For the group as a whole there was no effect of this kind. However, individuals differ in the degree of influence that others have on their music preference. This was evident from the between-subject variability of reviewer influence on ratings of song desirability in our present study (Figure S1). It is known that intersubject differences of nonsocial influence on value can be tracked by intersubject differences of neural activity when learning is occurring [5, 31, 32]. Consequently, we used a behavioral marker of tendency to be influenced by reviewer reviews, *B*_inf_, to weight our group analysis as a between-subject regressor. This allowed us to observe the effect of influence on value processing in the brain according to the degree to which influence is actually expressed in behavior. We predicted that influence-related activity in the brain would be high on this contrast when *B*_inf_ was high and low when *B*_inf_ was low. Confirming our predictions with a whole-brain cluster-corrected analysis, when a person had been exposed to an opinion about an object and was influenced by this exposure, the magnitude of the ventral striatum response to that object's value changed accordingly (Figure 4).

Opinions are just one of many factors that affect our valuation of objects. Nonsocial influences, such as the range of potential outcomes [6] and temporal discounting [7], affect value magnitude processing in the ventral striatum. Social comparison (where object value depends on what other people have received) influences reward value in the same region [8]. With our findings, it seems that both social and nonsocial influences affect valuation at the same basic level in the human brain.

In a computational account of valuation [33], sensory information about an object is considered inherently uncertain, forcing the subject to make inferences (e.g., Bayesian) from available information in the environment. In perception, a small white object might be perceived as an egg in an egg carton but as a ball on a golf course because the environment is awash with prior beliefs of what to expect. With value, reviewer opinions may also provide a source of prior information about the underlying worth of an object, consequently biasing the subject's valuation. This bias will occur if there is also a strong prior belief that a reviewer's review is a good indication of an object's worth.

The ventral striatum is ideally connected for updating values via inputs from hippocampus, amygdala, frontal cortex, and the mesolimbic dopamine pathway [34, 35]. Dopamine itself has a critical role in assigning value to objects [36–41]. Electrophysiological recordings have found that dopamine neurons can signal unexpected delivery or absence of rewards [42–44]. Among other information, this signal carries information about the reward's magnitude [45–47]. Critically, it signals the magnitude of reward relative to what is expected rather than on some absolute scale [48]—demonstrating that the dopamine system has the flexibility in value processing that would be necessary for social influences on value. Recent fMRI research has shown that the magnitude of reward signals in the human ventral striatum is also modulated by dopaminergic influences [9]. Taken together with our results, this suggests that dopamine might be mediating social influence on object value.

The question remains as to why the value of an object is increased or decreased with the opinion of reviewers. Object value could change with expectations of social consequences to owning it (e.g., approval of others, affiliation, positive self-image, social status). Alternatively, object value could change with expectations of nonsocial features of the song (e.g., sound quality). Experts could be assumed to have expertise in either case. To test the first case, one could measure expectations of social consequences of owning a song as a function of expert opinion and relate this to reward activity when receiving it. To test the latter, one might examine whether the song sounds different (or better or worse) to the subject on the basis of expert opinions or if its nonsocial qualities elicit different responses in the subject as a result of socially altered prior expectations.

#### Unanimous Opinions

We contrasted “unanimous agreement” review outcomes (both reviewers prefer the subject's preference) with “split” review outcomes (experts disagree with each other) (Figure 2C). We also contrasted “unanimous disagreement” review outcomes to split review outcomes (Figure 2D). In both comparisons, right anterior insula activity (bordering on lateral orbitofrontal cortex) was greater when the opinions of both reviewers were the same (R_S_ – R_SPLIT_ peak 34, 18, −14; R_A_ – R_SPLIT_ peak 42, 24, −8).

Anterior insula activity is often positively associated with uncertainty, and one would expect that split reviewer opinion would produce more uncertainty about an object's value or one's ability to choose rewarding music than a unanimous opinion. Thus, our finding was unexpected. One explanation could be that activity reflected updating of object values, rather than uncertainty. However, the number of unanimous reviews (relative to split reviews) that a song received did not predict the subject-reported change in that song's value (see Supplemental Experimental Procedures). Likewise, anterior insula activation was not proportional to the tendency for an individual to change song value (*B*_inf_) (see Supplemental Experimental Procedures).

A more likely explanation is that the anterior insula was representing current and predicted feeling states associated with the opinions of others [49, 50]. Future work could test whether subjects have stronger feeling states when facing unanimous agreement or disagreement from others. If this were the case, activity in this region may relate to why unanimous opinions are critical for normative influence (i.e., compliance, influenced to gain social approval without changes in private beliefs) [51, 52].

### General Discussion

Humans and animals use the reactions of others to help determine what is valuable: what to eat, what is dangerous, what is attractive, and (for humans) what to wear, what medicine to take, and for whom to vote—to give but a few examples. Each object, from food to parliamentary candidate, has a perceived value, which can be changed through social influence. Consequently, understanding how our values are changed by social influence is of considerable importance. We have shown that, when effective, the opinions of others alter a very basic mechanism of the human brain that reflects an immediate change in our values. Social influence at such a basic level may contribute to the rapid learning and spread of values throughout a population. These values could range from the quality of food to race and gender stereotypes. In a world where not everyone is influenced to the same degree, we are also a step closer to knowing whose values have changed through social contact, and to what extent, by observing a quantifiable physiological process.

## Supplemental Information

Document S1. Two Figures, Two Tables, and Supplemental Experimental Procedures
