# Supplementary material for: How the Opinion of Others Affects Our Valuation of Objects
Source: Curr Biol. 2010 Jul 13;20(13):1165–70. doi: 10.1016/j.cub.2010.04.055 (PMC2908235; doi:10.1016/j.cub.2010.04.055)
Supplement: Document S1. Two Figures, Two Tables, and Supplemental Experimental Procedures [file mmc1.pdf]

Current Biology, Volume *20*

Supplemental Information

How the Opinion of Others Affects Our Valuation of Objects

Daniel K. Campbell-Meiklejohn, Dominik R. Bach, Andreas Roepstorff, Raymond J. Dolan, and Chris D. Frith

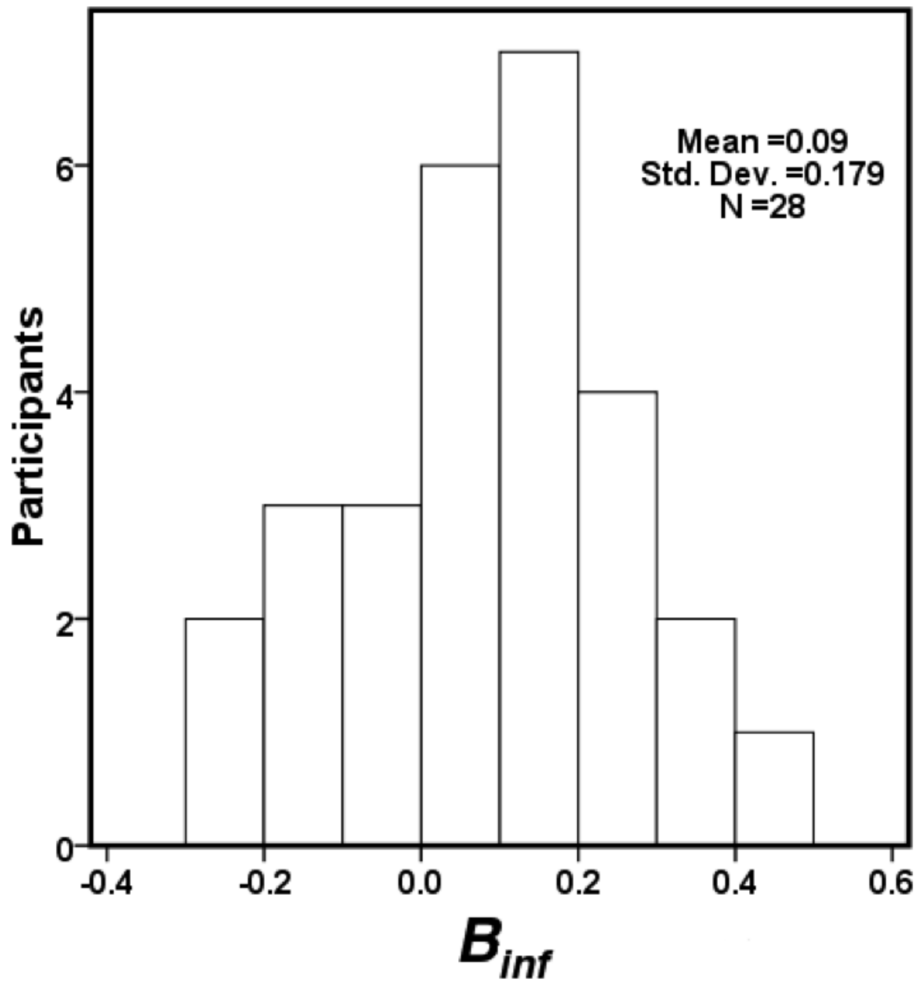

Figure S1. Frequency histogram (number of subjects) of B_{inf}. B_{inf} is the standardized beta coefficient from a linear regression that predicts the change in song valuations (in standard deviations) from net expert opinion of the song. B_{inf} was used to as a

between-subject covariate the group analysis of disagreement (Figure 3) and social influence on value (Figure 4).

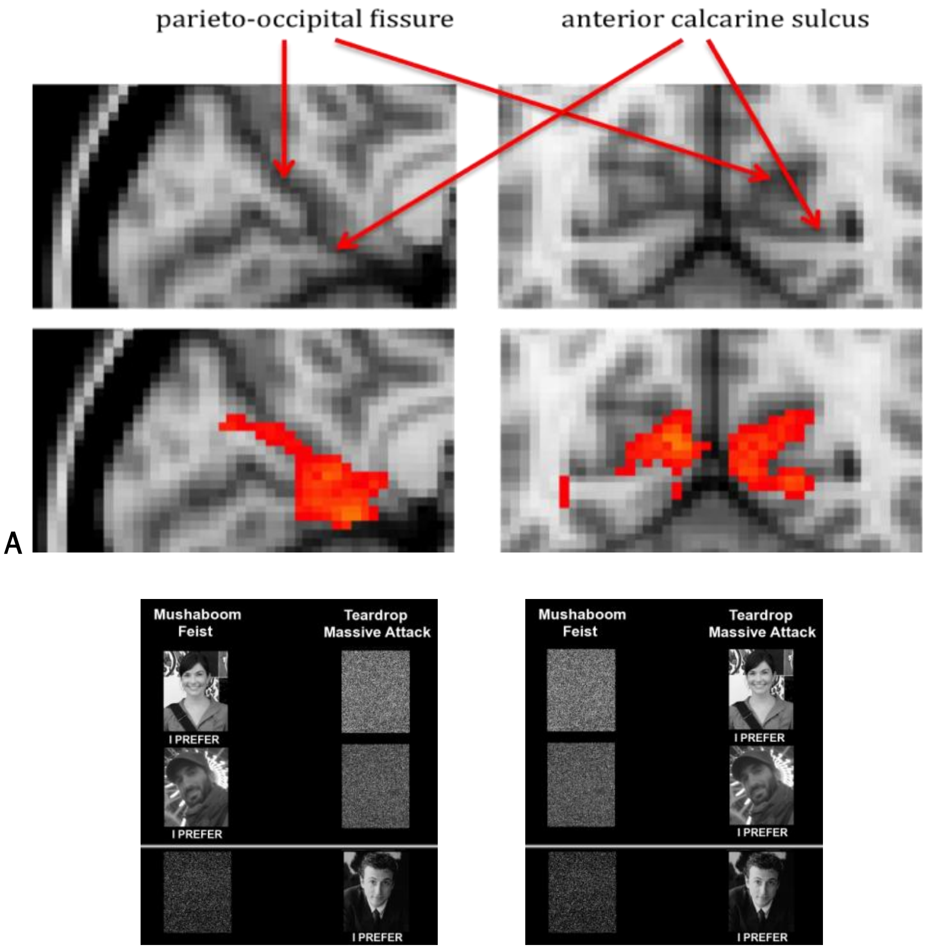

B. Reviewers Choose Alternative Song | Reviewers Choose Subject's Song

Figure S2. Posterior activation of review outcome. Panel A shows parieto-occipital fissure and anterior calcarine sulcus activation for review outcome (agreement > disagreement, $[R_S S+R_S A]-[R_A S+R_A A]$). Activations are whole-brain cluster corrected Z statistic maps ($Z > 2.3$, $p < 0.05$), which were overlaid onto the standard MNI brain. Due to the activation's location along a sulcus/fissure it is challenging to interpret. Peak activation can be assigned to either side of the sulcus/fissure, depending on which atlas is consulted (Table S2). The significant activation (observing the entire blob) follows the parieto-occipital fissure and anterior calcarine sulcus and extends into both posterior cingulate cortex and visual areas [1]. MNI coordinates (mm) of view in Panel A: x = -8; y = -60. Within the region of the anterior activation, prior meta-analyses of fMRI studies have revealed consistent activation during tasks of autobiographical memory, imagining oneself in the future and theory of mind [2], supporting a theory that this area mediates mental projection of the self to other

times, places and perspectives [3]. Self-referencing social stimuli such as being liked by others [4] and agreement/conformity with normative opinion [5] also activate this region. From this perspective, activation might have resulted from self-reference while evaluating agreement with others. On the other hand, the posterior areas of activation could be assigned to occipital regions, including intracalcarine cortex, lingual gyrus, and precuneus. Moreover, there was additional activation in what is clearly occipital cortex (peak (mm) -18 -86 -14) (Table S1). The Oxford-Harvard Cortical Structural Atlas and Jülich Histological Atlas [6, 7] place this most posterior activation in occipital fusiform cortex and V4. Activity within the visual system has been previously shown to be modulated by value [e.g. 8, 9, 10] and therefore may have been affected reward from agreement with reviewers. Stimulus "value" is but one of several known factors that bias activation levels within the regions of visual cortex that represent incoming sensory information from the visual field (see [8] for discussion on this topic). Overall, this increase in visual activation may represent mechanisms that bias spatially selective areas of visual cortex in favor of more valuable stimuli. Alternatively, this activation may relate to other attention-related processes. As Panel B shows, when reviewers preferred the alternative song, their pictures were moved to the opposite side of the display. In this case, subjects may have looked at both sides of the display (and both song tokens) more often than when reviewers preferred the same song token as the subject. Multiple visual stimuli has been previously shown to result in net suppression of BOLD activity in visual areas, across hemispheres and particularly in V4, as neighboring stimuli compete for neural representation [11-13]. This could therefore result in relatively more activity with reviewer agreement but would not, to our knowledge, affect the interpretation of other findings in the study.

Table S1. All fMRI activations: cluster size (voxels), Z score at peak voxel, peak MNI coordinates (mm), and associated anatomy from [1, 7] and the Oxford-Harvard structural atlases. All activations in table were cluster corrected with a voxel selection threshold (for clusters) of Z > 2.3 and cluster significance level of $p < 0.05$. Local pk = within same cluster as above.

fMRI Contrast	cluster size	Z_{max}	Peak Voxel			Anatomy
			x	y	z	
**object outcome** (preferred > non preferred) $[R_S S+R_A S]-[R_S A+R_A A]$	410	3.25	-46	40	4	lateral prefrontal cortex
	489	4.06	14	10	-8	ventral striatum
	376	3.47	-18	0	-16	amygdala
	local pk	3.23	-16	16	-2	ventral striatum
	309	3.58	-2	-36	34	posterior cingulate cortex
**review outcome** (agreement > disagreement) $[R_S S+R_S A]-[R_A S+R_A A]$	816	4.62	-10	8	-12	ventral striatum
	1118	3.64	8	-62	10	anterior calcarine sulcus / parieto-occipital fissure (see Table S2, Figure S2)
	local pk	3.61	-16	-56	2	as above
	480	3.23	-18<br>-8	-86<br>-86	-14<br>-18	occipital fusiform gyrus, V4 (see Figure S2)
**review outcome, only anti-influenced subjects (negative B_{inf})** (n = 7) (agreement > disagreement) $[R_S S+R_S A]-[R_A S+R_A A]$	487	3.78	-6	16	2	ventral striatum
	local pk	3.69	6	14	-6	
**review outcome x B_{inf}** (disagreement > agreement) x B_{inf} $[R_A S+R_A A]$-$[R_S S+R_S A]$ x B_{inf}	1785	3.94	-44	48	4	lateral prefrontal cortex
	1296	3.91	36	48	22	lateral prefrontal cortex
	542	3.53	4	16	34	dorsal anterior cingulate cortex
	711	4.04	-38	14	0	insula cortex
	761	3.87	52	8	2	central opercular cortex / insula cortex
	479	3.67	66	-30	36	temporoparietal junction
**object outcome & review outcome overlap**	84	4.2	-8	10	-10	ventral striatum
**review outcome x object outcome x influence** $[R_S S-R_S A]-[R_A S-R_A A]$ * B_{inf}	400	3.44	10	18	-8	ventral striatum
	local pk	3.33	-6	14	-8	
**unanimous expert agreement** $[R_S-R_{SPLIT}]$	721	3.88	34	18	-14	lateral orbitofrontal cortex / anterior insula cortex
**unanimous expert disagreement** $[R_A-R_{SPLIT}]$	430	4.01	42	24	-8	lateral orbitofrontal cortex / anterior insula cortex

Table S2. Parieto-occipital fissure / anterior calcarine sulcus activation for review outcome (agreement > disagreement). Due to the limited spatial resolution of fMRI and variation between different atlases, peak activation could be considered to be located in a variety of areas on both sides of the parieto-occiptial fissure and anterior calcarine sulcus. Locations of peak activations according to various brain atlases are indicated.

Peak voxel [MNI (mm)] coordinates Z_{max}	Oxford-Harvard Cortical Structural Atlas (FSL)	MNI Structural Atlas [14, 15]	Talairach Atlas [16-19]	Jülich Histological Atlas [6, 7]	Duvernoy Atlas [1]
8 -62 10<br>Z_{max} = 3.64	50% intracalcarine cortex<br>19% precuneus cortex<br>8% lingual gyrus<br>6% supracalcarine cortex	52% occipital lobe<br>15% parietal lobe	posterior cingulate cortex,<br>BA 30	82% V1, BA17<br>42% V2, BA18	cuneus
-16 -56 2<br>Z_{max} = 3.61	44% lingual gyrus<br>26% precuneus cortex<br>8% posterior cingulate cortex<br>1% intracalcarine cortex	67% parietal lobe<br>12% occipital lobe	lingual gyrus	81% V1 BA17<br>46% V2 BA18	superior lingual gyrus

SUPPLEMENTAL EXPERIMENTAL PROCEDURES

Subjects

Twenty-eight neurologically and psychologically healthy (15 males, 13 females; mean age 26.6 ± 5.24 (S.D.); age range 19 to 39, all right-handed) gave informed consent and participated in the study. Each was recruited via public advertisements in the London, UK. Subjects had normal or corrected-to-normal vision. The UCL Research Ethics Committee approved this study. Subjects received 20 British pounds sterling in guaranteed payment and 10 songs on a CD.

Pre-scanning

One week prior to scanning, subjects submitted a list of twenty songs that could be purchased from an online music store. Each was a song that the subject desired but did not yet own. On arrival to the centre, subjects had their photo taken and rated each of their 20 songs for desirability on a scale from 1 (I do not want this song) to 10 (I really want this song). Subjects also looked at pictures of two music 'experts' and read descriptions of the two them, as follows:

> Dave is a respected musician and sometimes London DJ. He has been listening to and playing music as long as he can remember. He owns a massive collection of music from over 50 countries, but he also listens to the top 40 at work. He is an avid drummer and plays guitar. When DJing, he creatively mixes samples from anything from hip hop to the Beatles and describes his music taste as "eclectic but with a good ear for quality sounds."
>
> Michelle is a music writer. Michelle is always listening to music. She reviews albums for UK and USA music magazines, interviews up-and-coming artists and often has access to music well before the general public. She describes

> her music taste as very open, and listens to a wide variety. She likes new and independent artists, but admits that she also listens pop music while out and about in town and with friends.

Subjects were asked to rate each reviewer from 1 (not at all) to 7 (very much) for how much the person could be trusted to pick music that the subject would like. No comparisons were made between experts during the study. Descriptions were created to communicate a degree of expertise across a broad range of popular music tastes.

Subjects were informed that the two experts had listened to the 20 songs and provided reviews for each. Reviews were preferences between each of the 20 subject-provided songs and an alternative song, provided by the experimenter. Each subject-provided song was reviewed six times (relative to six different alternative songs). Subjects received instructions for the task and answered a series of questions to confirm that their task was understood. Each subject confirmed that they believed the reviews were real.

Task and Timing

The task was programmed and run using Presentation v.12 (Neurobehavioural Systems). Visual displays were back-projected to a display in the scanner. Subjects viewed the displays via a mirror placed above their eyes. Responses (from the right hand) were collected using by two fibre-optic button boxes.

Each trial (see Figure 1) began with a choice for the subject. We presented subjects with two songs at the top of the screen. One was a song that the subject provided. The other was an alternative, provided by the experimenter. The alternative was a Canadian or Scandanavian pop song, which was real but unknown to the subject (confirmed after the scan session). Song choices were randomly assigned to the left and right side of the display. Pictures of the experts were arranged vertically down the centre of the display. A picture of the subject appeared at bottom of the screen, beneath the expert pictures. The words 'I prefer' were placed under each photo.

The subject's task was to move their own picture beneath the song they desired the most. Subjects pressed the left button to move their picture left, or the right button to move it right. A scrambled picture of the subject was placed under the song they did not choose. Subject-provided songs appeared equally-often on left and right sides of the display. Subjects were told that the song that they chose had a slightly (less than 5%) higher chance of being chosen for a token at the end of the trial to provide motivation to pick their real preference. Each song actually had a 50% chance of being chosen. Subjects knew that the songs with the most tokens at the end of the task were to be purchased for them and placed on a CD. There was a time limit of 2 seconds to make a choice. If no choice was made, a large 'X' appeared on the screen for the remainder of the trial.

After making their choice, subjects learned about the expert's opinions. The pictures of each expert were moved under their respective preference. Scrambled pictures of the experts were placed under songs they did not choose. Experts could both prefer

the subject-provided song, both prefer the alternative, or disagree with each other. This phase is termed the 'review outcome.' Next, the songs alternately changed color between green and white (every 50ms, for 1s). Finally, a song was chosen for a token and appeared at the bottom of the screen. This phase was the 'object outcome.'

Review outcomes were completely independent from object outcomes. During instruction, subjects confirmed that expert choices did not predict which song token would be received. The subject received a token for each of their submitted songs as often as they received an alternative. The order of trials was optimized to provide maximum efficiency for detection of Blood Oxygenation Level Dependent (BOLD) activity related, independently, to different review and object outcomes. For these purposes, it was not possible to use real expert reviews, and confederate reviews were used in their place. Each participant confirmed that they believed the reviews were real. As a result, trials could be placed close together in time with a brief minimum of 3 seconds between each modeled event (see section on fMRI analysis) reducing subject time in the scanner but still controlling for nonlinearities of the BOLD signal [20].

Decisions appeared at time 0 of each trial. Review outcomes appeared at 3 seconds, and songs began to flash at 4 seconds. Object outcomes were presented at 5 seconds and remained on display for 2 seconds. A fixation cross was displayed for 2 seconds between each trial.

There is no non-social equivalent, to our knowledge, to a human opinion. Even a 'computer' from which one could make accurate inferences of subjective human value only acts as an indirect inference of the human opinions used to program it, and thus computer reviews would still remain 'social.' For this reason, we saw little merit in providing an artificial 'non-social' control in this study.

Post-scanning

After completing the task, subjects rated each of their 20 songs for desirability for a second time. Subjects were also asked if they had learned more about the reviewers or more about the songs. The 10 songs for which the subject had the most tokens (from the object outcome of the task) were purchased for the subject.

Conditions

Only trials in which subjects chose the same song as they had provided a week prior were included in the analysis to prevent analysis of subject errors. Key independent variables were:

1. **Review outcome**
   - i. R_S (experts chose the subject-preferred song)
   - ii. R_A (experts chose the alternative)
   - iii. R_{SPLIT} (split; one expert chose the subject-preferred song; the other chose the alternative).

2. **Object outcome:**
   - i. S (subject gains a token for their preferred song)
   - ii. A (subject gains a token for the alternative song).

These variables formed a 2*3 design matrix (Figure 1) of six experimental conditions: **R_SS, R_SA, R_AS, R_AA, $R_{SPLIT}S$** and **$R_{SPLIT}A$.**

Behavioural Analysis

Behavioral analysis was completed using SPSS Statistics 16.0. Net expert opinion (number of R_S – number of R_A) was calculated for each subject-preferred song. Change in song value was calculated as the difference between post-scan and pre-scan ratings for desirability. For each subject, a linear regression was carried out to determine the effect of the net expert opinion on change in song value. This provided a standardized beta coefficient, B_{inf}, for each subject – representing the degree (in standard deviations) to which value of songs increased or decreased with expert opinion (see Figure S1). B_{inf} was used as a between-subject regressor for subsequent fMRI analysis. A linear regression was also performed to test the effect of unanimous review frequency (which varied from 4 to 7 per song) on change in a song value. Gender effects on B_{inf} were tested with an independent samples t-test.

Functional Magnetic Resonance Imaging (fMRI)

Standard fMRI acquisition, and preprocessing were used in this study.

Acquisition

Scanning took place at the Wellcome Trust Centre for Neuroimaging in London, UK. Subjects were scanned at 3 Teslas with a Siemens MAGNETOM Trio scanner (Siemens Medical Solutions, Erlangen, Germany) fitted with a 12-channel head coil. Field maps were acquired with a standard double echo gradient echo field map

sequence (TE, 10.0 and 12.46 ms), using 64 slices covering the whole head (voxel size, 3*3*2 mm with 1mm gap between slices). Functional data was collected as T2-weighted echo planar images (EPI) in descending slice acquisition order. Each volume (voxel size: 3*3*3mm; TE, 30 ms; TR, 3360ms) contained 48 slices, covering the whole brain. BOLD sensitivity losses in the orbitofrontal cortex due to susceptibility artifacts were minimized by applying a z-shim gradient moment of -1.4 mT/m*ms, a slice tilt of -30°, and a positive PE gradient polarity [21]. 176-slice whole-brain anatomical scans (matrix, 256*256; 1mm slice thickness; TE, 2.48ms; TR=7.92ms; flip angle, 16°; TI=910ms) were acquired using a modified driven equilibrium Fourier transform (MDEFT) sequence with optimized parameters as described previously [22] for co-registration with the EPI data. Images were reconstructed by performing a standard 3D Fourier Transform, followed by modulus calculation. No data filtering was applied in k-space or in the image domain.

Preprocessing

Image unwarping, and motion correction was performed using statistical parametric mapping (SPM 5; Wellcome Trust Centre for Neuroimaging; www.fil.ion.ucl.ac.uk/spm) on Matlab (version 7.1, MathWorks). EPI images were generated off-line from the complex k-space raw data using a generalized reconstruction method based on the measured EPI k-space trajectory to minimize ghosting. They were then corrected for geometric distortions caused by susceptibility-induced field inhomogeneities. A combined approach was used which corrects for both static distortions and changes in these distortions due to head motion [23, 24]. The static distortions were calculated for each subject from a field map that was processed using the FieldMap toolbox as implemented in SPM5. Using these

parameters, the EPI images were then realigned and unwarped with a procedure that allows the measured static distortions to be included in the estimation of distortion changes associated with head motion. The remaining preprocessing and was carried out with the FMRIB's Software Library (FSL) version 5.63 [25]. Brain matter was segmented from non-brain using a mesh deformation approach [26]. High pass temporal filtering was applied using a Gaussian-weighted running lines filter, with a cut-off of 50s [27]. Each volume was smoothed with a Gaussian filter (full-width half-maximum of 5mm). Independent Component Analysis was used to visually identify and remove artifacts in the data using Multivariate Exploratory Linear Optimized Decomposition into Independent Components (MELODIC) software [28].

Single Subject General Linear Models and fMRI Analysis

Modeling and statistical analysis of fMRI data was carried out with the FEAT (FMRI Expert Analysis Tool, www.fmrib.ox.ac.uk/fsl) version 5.63 [25]. A standard general linear model (GLM) was used for individual subject analyses. The GLM was fit in pre-whitened data space (to account for autocorrelation in the FMRI residuals [29]). Regressors corresponding to each condition of the 2*3 design matrix (Figure 1) (plus their temporal derivatives) were included in the model as stick functions placed midway through the 'object outcome' display period. Decisions, trials in which subjects took longer than 2s to respond, and trials in which the subject chose the alternative song (i.e. not their pre-submitted song) were included in the model as independent regressors but not used in further analysis. Regressors were convolved with the FSL default haemodynamic response function (HRF, gamma function, delay = 6s, standard deviation = 3 s). High-pass temporal filtering (50s) was also applied to

the regressors. GLM results were estimated [30] and transformed, after spatial normalization, into standard (MNI152) space [27].

Single Subject Contrasts

The following contrast images (and their inverse contrasts) were generated from the GLMs.

1. **Review Outcome [R_SS + R_SA] – [R_AS + R_AA]:** Outcomes when both experts agreed with the subject preference compared to outcomes in which both experts preferred the alternative song.
2. **Object outcome [R_SS + R_AS] – [R_SA + R_AA]:** Outcomes when the subject received the song they preferred compared to when they received the alternative.
3. **Review Outcome x Object outcome [R_SS – R_SA] – [R_AS – R_AA]:** Influence of expert opinion on song value activity.
4. **Unanimous Expert Agreement [R_S] – [R_{SPLIT}]:** Both experts agree with the subject compared to one choosing the subject's song and the other choosing the alternative.
5. **Unanimous Expert Disagreement [R_A] – [R_{SPLIT}]:** Both experts disagree with the subject compared to one choosing the subject's song and the other choosing the alternative.

Group-level Analysis

Group-level analysis was carried out with FLAME 1+2 (FMRIB's Local Analysis of Mixed Effects [30]. All subjects were modeled as a single group. A GLM was fit to

the effects of the contrasts described above. This was done in two separate group analyses:

1. Group mean
2. Group mean + a between subject regressor of B_{inf}

All Z statistic maps were cluster corrected (contiguous clusters defined by $Z > 2.3$) with a whole brain cluster significance level of $p < 0.05$ [31-33]. To note the sub-threshold bilateral effect of 'review outcome' in the ventral striatum (Figure 2B), this contrast was also analyzed with contiguous clusters defined by voxels $Z > 2.0$, $p < 0.05$ (cluster corrected).

Further Investigation into the Nature of Ventral Striatum Responses to Agreement

The fact that some of the individuals had a negative B_{inf} (Figure S1) and changed their value of songs in the opposite direction to net reviewer opinion made the mean group activation in ventral striatum with respect reviewer agreement more challenging to interpret as a reward response. If the ventral striatum signaled a reward with agreement, one could propose that the effect would be stronger or perhaps only present in those subjects with positive B_{inf} values (i.e. those subjects whose opinions of the songs conformed to the opinions of reviewers). This is based on the assumptions that (a) if subjects conform their opinions to those of reviewers, their motivation is derived from the presence of a reward from agreement and (b) those that do not conform do not experience a reward from an agreement. We tested if those with negative B_{inf} values still produced a ventral striatum response with agreement with experts (whole brain, $Z > 2.3$, $p < 0.05$ cluster corrected). Seven participants reduced their subjective value of a song as the number of positive

reviews of that song increased (negative B_{inf}). Group analysis using just these subjects still produced a ventral striatum response to 'review outcome' [R_SS+R_SA]–[R_AS+R_AA] (right peak: 6, 14, -6; left peak -6, 16, 2) (Table S1). This result is discussed in the main text.

Testing the Potential Impact of Unanimous Reviews on Object value

Lateral orbitofrontal cortex / anterior insula cortex activity during unanimous reviewer opinion relative to split opinions might be interpreted as mediating the impact of these unanimous reviews on song values. To test this interpretation, we completed regression analyses to see if the subject's change in song value (and, separately, the absolute value of that change in song value) varied as (a) a function of the number of unanimous reviews received for each song and (b) a function of the net reviewer opinion of the song weighted by the number of unanimous reviews that made up that net reviewer opinion. No significant effect of unanimous reviews were observed in either case ($Fs(1,19) < 0.53$, p's > 0.47). We also tested if BOLD activation from unanimous review outcomes (relative to split review outcomes; single subject contrasts 4 and 5) varied between subjects with tendencies to be influenced by reviewer opinion (B_{inf}). Again, no significant relationship was observed. These results are discussed in the main text.

Testing the Relationship between Disagreement Activity and Influence Activity

We tested if activations during disagreement with the experts (relative to agreement) (Figure 3) predicted changes of ventral striatum activity due to social influence on object value (Figure 4) in the same subjects. Each active cluster correlating with B_{inf} during disagreement was converted to a mask. These masks (right insula / central opercular cortex, left insula, dorsal anterior cingulate cortex, left prefrontal cortex, right prefrontal cortex, temporoparietal junction) were used to calculate the percent signal change within each of these regions, for each participant, during disagreement with the experts (relative to agreement with experts). The same was done to calculate the percent signal change for each subject within the area defined by Figure 4's cluster resulting from the interaction between review outcome and object outcome activity (influence). We then completed a regression analyses to see if the percent signal change within any cluster during disagreement with reviewers predicted the percent signal change in ventral striatum due to social influence on object value. No significant relationships were observed.

Gender Effects

No significant effect of gender was observed on susceptibility to influence (B_{inf}) ($t(26) = 0.647$; $p < 0.543$). Likewise, no effect of gender was observed in reported BOLD activations when entered as a between-subject factor.

Supplemental References

1. Duvernoy, H. (1999). The Human Brain Surface, Blood Supply, and Three Dimensional Sectional Anatomy, 2nd Edition, (New York: Springer-Verlag Wien NewYork).
2. Spreng, R.N., Mar, R.A., and Kim, A.S. (2009). The common neural basis of autobiographical memory, prospection, navigation, theory of mind, and the default mode: a quantitative meta-analysis. J Cogn Neurosci *21*, 489-510.
3. Buckner, R.L., and Carroll, D.C. (2007). Self-projection and the brain. Trends Cogn Sci *11*, 49-57.
4. Davey, C.G., Allen, N.B., Harrison, B.J., Dwyer, D.B., and Y¸cel, M. (2009). Being liked activates primary reward and midline self-related brain regions. Human brain mapping *9999*, NA.
5. Klucharev, V., Hytonen, K., Rijpkema, M., Smidts, A., and Fernandez, G. (2009). Reinforcement learning signal predicts social conformity. Neuron *61*, 140-151.
6. Eickhoff, S.B., Paus, T., Caspers, S., Grosbras, M.H., Evans, A.C., Zilles, K., and Amunts, K. (2007). Assignment of functional activations to probabilistic cytoarchitectonic areas revisited. Neuroimage *36*, 511-521.
7. Eickhoff, S.B., Stephan, K.E., Mohlberg, H., Grefkes, C., Fink, G.R., Amunts, K., and Zilles, K. (2005). A new SPM toolbox for combining probabilistic cytoarchitectonic maps and functional imaging data. Neuroimage *25*, 1325-1335.
8. Serences, J.T. (2008). Value-based modulations in human visual cortex. Neuron *60*, 1169-1181.
9. Fliessbach, K., Weber, B., Trautner, P., Dohmen, T., Sunde, U., Elger, C.E., and Falk, A. (2007). Social comparison affects reward-related brain activity in the human ventral striatum. Science *318*, 1305-1308.
10. Shuler, M.G., and Bear, M.F. (2006). Reward Timing in the Primary Visual Cortex. Science *311*, 1606-1609.
11. Fink, G.R., Driver, J., Rorden, C., Baldeweg, T., and Dolan, R.J. (2000). Neural consequences of competing stimuli in both visual hemifields: a physiological basis for visual extinction. Ann Neurol *47*, 440-446.
12. Kastner, S., De Weerd, P., Desimone, R., and Ungerleider, L.G. (1998). Mechanisms of directed attention in the human extrastriate cortex as revealed by functional MRI. Science *282*, 108-111.
13. Kastner, S., and Ungerleider, L.G. (2000). Mechanisms of visual attention in the human cortex. Annu Rev Neurosci *23*, 315-341.
14. Mazziotta, J., Toga, A., Evans, A., Fox, P., Lancaster, J., Zilles, K., Woods, R., Paus, T., Simpson, G., Pike, B., et al. (2001). A probabilistic atlas and reference system for the human brain: International Consortium for Brain Mapping (ICBM). Philos Trans R Soc Lond B Biol Sci *356*, 1293-1322.
15. Collins, D.L., Holmes, C.J., Peters, T.M., and Evans, A.C. (1995). Automatic 3-D model-based neuroanatomical segmentation. Human brain mapping *3*, 190-208.

16. Laird, A.R., Robinson, J.L., McMillan, K.M., Tordesillas-Gutierrez, D., Moran, S.T., Gonzales, S.M., Ray, K.L., Franklin, C., Glahn, D.C., Fox, P.T., et al. (2010). Comparison of the disparity between Talairach and MNI coordinates in functional neuroimaging data: validation of the Lancaster transform. Neuroimage *51*, 677-683.
17. Lancaster, J.L., Tordesillas-Gutierrez, D., Martinez, M., Salinas, F., Evans, A., Zilles, K., Mazziotta, J.C., and Fox, P.T. (2007). Bias between MNI and Talairach coordinates analyzed using the ICBM-152 brain template. Human brain mapping *28*, 1194-1205.
18. Lancaster, J.L., Woldorff, M.G., Parsons, L.M., Liotti, M., Freitas, C.S., Rainey, L., Kochunov, P.V., Nickerson, D., Mikiten, S.A., and Fox, P.T. (2000). Automated Talairach atlas labels for functional brain mapping. Human brain mapping *10*, 120-131.
19. Talairach, J., and Tournoux, P. (1988). Co-Planer Stereotaxic Atlas of the Human Brain, (New York: Thieme).
20. Friston, K.J., Zarahn, E., Josephs, O., Henson, R.N., and Dale, A.M. (1999). Stochastic designs in event-related fMRI. Neuroimage *10*, 607-619.
21. Weiskopf, N., Hutton, C., Josephs, O., and Deichmann, R. (2006). Optimal EPI parameters for reduction of susceptibility-induced BOLD sensitivity losses: a whole-brain analysis at 3 T and 1.5 T. Neuroimage *33*, 493-504.
22. O'Doherty, J., Dayan, P., Schultz, J., Deichmann, R., Friston, K., and Dolan, R.J. (2004). Dissociable Roles of Ventral and Dorsal Striatum in Instrumental Conditioning. Science *304*, 452-454.
23. Andersson, J.L., Hutton, C., Ashburner, J., Turner, R., and Friston, K. (2001). Modeling geometric deformations in EPI time series. Neuroimage *13*, 903-919.
24. Hutton, C., Bork, A., Josephs, O., Deichmann, R., Ashburner, J., and Turner, R. (2002). Image distortion correction in fMRI: A quantitative evaluation. Neuroimage *16*, 217-240.
25. Smith, S.M., Jenkinson, M., Woolrich, M.W., Beckmann, C.F., Behrens, T.E., Johansen-Berg, H., Bannister, P.R., De Luca, M., Drobnjak, I., Flitney, D.E., et al. (2004). Advances in functional and structural MR image analysis and implementation as FSL. Neuroimage *23 Suppl 1*, S208-219.
26. Smith, S.M. (2002). Fast robust automated brain extraction. Human brain mapping *17*, 143-155.
27. Jenkinson, M., Bannister, P., Brady, M., and Smith, S. (2002). Improved Optimization for the Robust and Accurate Linear Registration and Motion Correction of Brain Images. NeuroImage *17*, 825-841.
28. Beckmann, C.F., and Smith, S.M. (2004). Probabilistic independent component analysis for functional magnetic resonance imaging. IEEE Trans Med Imaging *23*, 137-152.
29. Woolrich, M.W., Ripley, B.D., Brady, M., and Smith, S.M. (2001). Temporal Autocorrelation in Univariate Linear Modeling of FMRI Data. NeuroImage *14*, 1370-1386.
30. Woolrich, M.W., Behrens, T.E.J., Beckmann, C.F., Jenkinson, M., and Smith, S.M. (2004). Multilevel linear modelling for FMRI group analysis using Bayesian inference. NeuroImage *21*, 1732-1747.

31. Friston, K.J., Worsley, K.J., Erackowiak, R.S.J., Mazziotta, J.C., and Evans, A.C. (1994). Assessing the significance of focal activations using their spatial extent. Human brain mapping *1*, 214-220.
32. Forman, S.D., Cohen, J.D., Fitzgerald, M., Eddy, W.F., Mintun, M.A., and Noll, D.C. (1995). Improved assessment of significant activation in functional magnetic resonance imaging (fMRI): Use of a cluster-size threshold. Magnetic Resonance in Medicine *33*, 636-647.
33. Worsley, K., Marrett, S., Neelin, P., Vandal, A.C., J., F.K., and A.C., E. (1996). A unified statistical approach for determining significant signals in images of cerebral activation. Human brain mapping *4*, 58-73.
